# Pore structure and its impact on susceptibility to coal spontaneous combustion based on multiscale and multifractal analysis

**DOI:** 10.1038/s41598-020-63715-z

**Published:** 2020-04-28

**Authors:** Zhang Yu, Zhang Xueqing, Yang Wen, Xin Haihui, Hu Sherong, Song Yu

**Affiliations:** 1 College of Geoscience and Surveying, China University of Mining & Technology, Beijing, 100083 China; 2 Key Laboratory of Coalbed Methane Resource & Reservoir Formation Process, Ministry of Education, China University of Mining & Technology, Xuzhou, 221116 China; 3 School of Earth Science and Engineering, Hebei University of Engineering, Handan, 056038 China; 4 School of Safety Engineering, China University of Mining & Technology, Xuzhou, 221116 China

**Keywords:** Biogeochemistry, Nanoscience and technology

## Abstract

The relationship between the properties of coal and its tendency to spontaneous combustion is critical for the environment, safety concerns, and economy. In this study, to eliminate the complex influence of moisture; the samples having similar moisture content were selected from Shanxi and Henan provinces. The chemical properties, physical properties, and tendency of coal samples to spontaneous combustion were characterized based on the conventional analysis, mercury intrusion porosimetry, fractal dimensions, and crossing point temperature (CPT). The results confirmed that the coal rank, volatile matter, oxygen contents, and fixed carbon content had a good linear relationship with the CPT. The relationship between the ash content and CPT presented a “U-shaped” non-linear correlation. For the pore size distribution, the total pore volume also possessed a linear positive correlation with the CPT. The fractal curves could be distinctly divided into two stages: low-pressure (<20 MPa) and high-pressure (>20 MPa), from which the fractal dimensions were obtained using the Sponge and Sierpinski models. The relationship between the fractal dimensions (*D*_*s*1_, *D*_*s*2_, and *D*_*g1*_) and CPT could be divided into two distinct stages: a decrease in the CPT with increasing fractal dimensions (2.6–2.85), and then an in increase in the CPT. CPT decreased with increasing parameters of *D*_*1*_, *D*_*2*_, *H*, and *D*_10_, and it gradually increased with increasing *D*_*-*10_*-D*_*1*0_, *D*_*-1*0_*-D*_*0*_, and *D*_*0*_*-D*_10_. The above characteristics are important to comprehensively and systematically reveal the mechanism of spontaneous combustion.

## Introduction

Low temperature (i.e., <200 °C) chemical and/or physical processes in fossil fuels result in the accumulation of heat, which thereby leads to spontaneous combustion^1,2^. Numerous problems are caused by spontaneous combustion of coal, such as environment issues (emission of greenhouse gases, toxic and harmful gases, and trace elements)^3–6^, safety concerns (dust and gas explosions, unstable overlying rocks, large cracks, and subsidence)^7–9^, and economical loss (destruction of apparatus and equipment, and loss of coal resources)^3,10^. Therefore, it is important to evaluate the degree of proneness for the prevention of the spontaneous combustion of coal.

The crossing point temperature (CPT) is an index to evaluate the tendency of coal to spontaneous combustion^11–13^. Coal properties, oxygen, and ignition are three main factors for predicting the spontaneous combustion tendencies of different coals in chemical and/or physical processes^14^. Numerous studies have been reported on the intrinsic properties of coal and its spontaneous combustion tendency^15–20^. Qi *et al*.^21^ analyzed the relationship between the CPT and moisture, coal rank, and sulfur content. Nimaje and Tripathy^22^ demonstrated that the parameters of the ultimate analysis had a significant correlation with the Olpinski index, and this relationship could be used as a reliable index to assess the susceptibility of Indian coals to spontaneous combustion. Chandra and Prasad^23^ found that low coal horizons (less than 36% V.M.; Romax. 0.90–1.25%) were least susceptible to spontaneous combustion. Pattanaik *et al*.^24^ demonstrated that the intrinsic properties of coal (stratigraphy or coal rank, volatile matter, and petrography) had a good relationship with the susceptibility indices of spontaneous combustion (CPT, and differential thermal analysis/DTA). Nimaje *et al*.^13^ analyzed the statistical relationship between the CPT and the proximate analysis parameters, and found that mixture surface regression (MSR) model was more effective in predicting spontaneous heating liability of coal.

The influence of water on the self-heat process is complex^25^. Moisture can accelerate the oxidation, and enhance the thermal response by hindering the formation of stabilized radicals^25^. However, the liability of spontaneous combustion of air-dried coal samples increases in the moisture content^18^. Zhang *et al*.^26^ and Qi *et al*.^21^ found that moisture may play a role in slowing down spontaneous combustion.

Several pore classification standards have been proposed, which are as follows: 1) Ходот^27^ classified pores into micropores (<10 nm in diameter), transitional-pores (10–100 nm), mesopores (100–1000 nm), and macropores (>1000 nm); the International Union of Pure and Applied Chemistry (IUPAC) has classified pores into micropores (<2 nm), mesopores (2–50 nm) and macropores (>50 nm); and Yao *et al*.^28^ classified pores into adsorption-pores (<100 nm) and seepage-pores (100 nm).

Pore structures play an important role in the combustion and oxidation of coal^1,29–31^. Pores provide a good pathway to the transport oxygen to coal surface at low temperature, which increases consumption of oxygen^32,33^. Small space or volume can improve the material performance^34^. Karsner and Perlmutter^35^ found that coal with a large pore volume had high oxidation rate. Parsa *et al*.^1^ evaluated the effect of densification on brown coal on its physical properties and spontaneous combustion propensity. The results exhibited that the decrease in micropore volume led to a decrease in the contact between oxygen and the coal surface, thereby the increasing the critical ignition temperature. Air can more easily contact the macropore structures of coal (the decreased in the reactivity is initially rapid) than the micropore structures^30^. Mainly investigations on coal structural characteristics such as the pore volume, surface area, pore size distribution, and heterogeneity are conducted to reveal the spontaneous mechanism.

Pore structure characteristics have been widely applied in Coalbed Methane (CBM) exploration and exploitation^36–39^, shale gas reservoirs, and predicting favorable zones^40–42^. Mono fractal approaches have been extensively used in investigating the fractal characteristics of pore structures^28,43,44^. However, a single-scale (mono-fractal) analysis or a single fractal dimension cannot explain the differences in the pore size intervals and types of erratic variation or local variation that occur in the inner distribution of pore sizes^45^. Therefore, a multi-scale fractal model is used for different coal ranks, and tectonically deformed coals with the same coal rank^37,46^. Besides, multifractal methods are also used for non-homogeneous porous media to reveal the heterogeneous pore characteristic^47–51^. Li *et al*.^51^ and Song *et al*.^45^ conducted a the multifractal analysis to investigate the variability and heterogeneity of tectonically deformed coals by high-pressure mercury intrusion experiment. Therefore, multifractal analysis is an efficient method to investigate pore size distribution. Nevertheless, focus on the relationship between the spontaneous combustion characteristics and heterogeneity in different coal ranks is lacking.

The objective of this study was to develop an understanding of the relationship between the properties of coal and its tendency to spontaneous combustion. The experiments were conducted by conventional analysis, mercury intrusion porosimetry, and CPT. To eliminate the complex influence of moisture in the process of spontaneous combustion, coal samples having similar moisture contents were selected. This was done to reveal the coal intrinsic properties (coal rank, volatile matter, oxygen contents, fixed carbon contents, and sulfur content), pore characteristics (pore volume, surface area, pore size distribution, and heterogeneity), and tendency of coal to spontaneous combustion. Both multiscale and multifractal methods were used to systematically investigate the heterogeneity and susceptibility to coal spontaneous combustion. Such research is important to comprehensively and systematically reveal the mechanism of spontaneous combustion.

## Experiments and Modelling

### Geological setting

**Figure 1:**
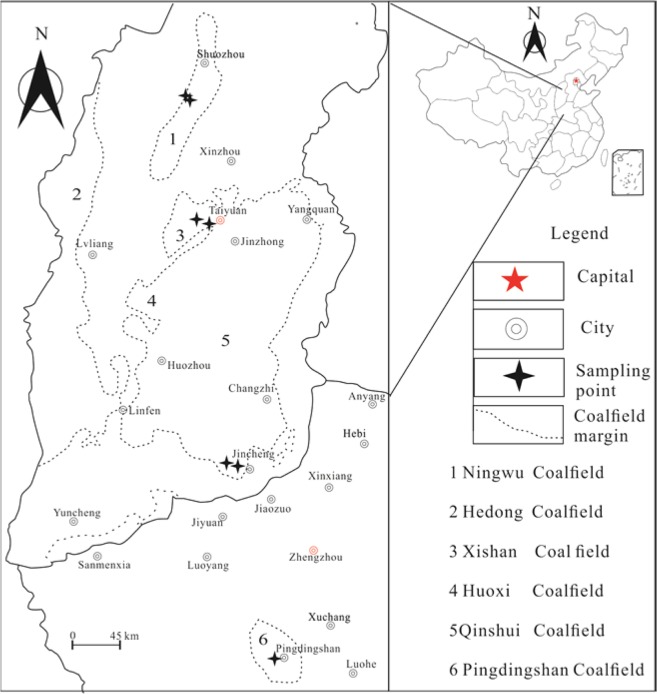
Positions of the research areas and the distribution of the sampling points.

A total of seven coal samples were collected from Shanxi and Henan provinces (Fig. 1), which contain the major coalfields in China. Six samples were collected from three coalfields in Shanxi, including Ningwu, Xishan, and Qinshui, and one sample was collected from Henan coalfield (Pingdingshan). The main coal-bearing stratum of these coalfields belongs to the Carboniferous–Permian system. Samples were directly collected from coal colliery following the Chinese Standard Method GB/T 19222–2003 and were carefully packed and taken to the laboratory for experiments.

### Samples and experiments

Seven coals were collected from Ningwu, Dongqu, Guandi, Longle, Fenghuangshan, and Pingdingshan collieries in Shanxi and Henan provinces. The coal samples were crushed to −200 meshes (~0.074 mm) and vacuum-dried. The ultimate analysis and proximate analysis were conducted in accordance with the Chinese national testing standards (GB/T 212–2008 and GB/T 31391–2015). Vitrinite random reflectance (%Rr) were measured for all the coal samples on the same polished sections using a Leitz MPV-3 photometer microscope, following conventional methods in accordance with the Chinese national standard (GB/T 6948–1998).

The CPT measurements were conducted at the China University of Mining and Technology. The details of the experiment are reported in our previous work^26^. The samples (mass 50 g with a particle size of ~0.18–0.43 mm) were exposed to a dry air flow of 50 mL/min within the reactor with a temperature ramp rate of 1 °C/min with coal and oven temperatures being recorded. When the coal temperature is equal to the oven temperature, the sample is at the CPT^21,52^.

The coal samples were prepared by a vacuum drying for 12 h at 70–80 °C. High pressure mercury injection (HPMI) experiment was performed for the samples using an Auto Pore IV 9510 HPMI instrument at the China University of Mining and Technology. The mercury injection pressure ranged from 0.90 to 4.0 × 10^4^ PSIA. The pore diameters obtained were from 5.35 to 2.28 × 10^5^ nm.

### Fractal dimensions

#### Sierpinski model

Based on the Sierpinski model^53^, the fractal dimensions (*D*_*s*_) can be calculated by the following equation:

1 $$ln(V)=(3-Ds)ln(P-Pt)+ln\alpha $$

where *V* is the mercury injection amount at *P* in mL, *D* is the volume fractal dimension, *P*_t_ is the mercury inlet pressure in MPa, and *α* is a constant. If the slope of the *ln(P* − *P*_*t*_*) vs. ln (V)* curve is *K*, then *D*_*s*_ is *3* − *K*.

#### Sponge model

Based on the Menegr model^53^, the fractal dimensions (Dg) can be calculated by the following equation:

2 $$Dg=ln[dVP(r)/dP(r)]-ln\alpha /lnP(r)+4,$$

where *V*_*P(r)*_ is the cumulative injection volume at a given pressure *P(r)* and *α* is a constant. Therefore, the pore fractal dimension, *D*_*g*_, can be obtained by: *D*_*g*_ *= 4* + *A*, where A is the slope in Eq. (2).

#### Multifractals

Multifractal analysis is used to measure the statistic Hg pore size distributions. The pore diameter interval (*I*) ranging from 0.006 nm to initial diameter (responding to the least pressure) was selected to generate a box (*N*(*ε*) = 2^*k*^, ε = L × 2^*k*^) by dyadic partitions in k stages (*k* = 1, 2, 3, …), where *L* is the length of the support^45,48,50,51^. The probability, *P*_j_(ε), of the pore size can be calculated as^48^,

3 $${P}_{j}(\varepsilon )=\frac{{N}_{j}(\varepsilon )}{{N}_{t}},$$

where, *N*_*j*_ (ε) is the volume of a box (*j*=1, 2, 3…), and *N*_*t*_ is the total volume of the system.

The probability for each box of size ε unit can be calculated as^48^,

4 $$P(\varepsilon )={\varepsilon }^{-aj},$$

where *α*_*j*_ is the coarse Hölder or singularity exponent for the boxes, which theoretically represents how the singularities of a system tend to infinity in the limit ε → 0.

The α exponent, *N*_*α*_, was used to evaluate the number of boxes, as follows:

5 $$N\alpha (\varepsilon ){\alpha }^{-f(\alpha )},$$

where the set of *f*(α) values represents the spectrum of fractal dimensions that characterize the abundance of the set of points with singularity α. *f*(α) can be calculated as^48^:

6 $$a(q)\propto \frac{{\sum }_{j=1}^{N(\varepsilon )}{u}_{j}(q,\varepsilon )log\{{p}_{j}(\varepsilon )\}}{log(\varepsilon )}$$

and

7 $$f\{\alpha (q)\}\propto \frac{{\sum }_{j=1}^{N(\varepsilon )}{u}_{j}(q,\varepsilon )log\{{u}_{j}(q,\varepsilon )\}}{\log (\varepsilon )},$$

where *μ*_*j*_ *(q, ε)* and *P*_*j*_*(ε)* are the normalized measures, defined as^48^:

8 $${\mu }_{j}(q,\varepsilon )=\frac{{p}_{j}^{q}(\varepsilon )}{{\sum }_{j=1}^{N(\varepsilon )}{\sum }_{j}^{p}(\varepsilon )}$$

9 $$\chi (q,\varepsilon )=\mathop{\sum }\limits_{j=1}^{N(\varepsilon )}{p}_{j}^{q}(\varepsilon ),$$

where *χ(q, ε)* can be calculated as:

10 $${\rm{\chi }}(q,\varepsilon )\propto {\varepsilon }^{-\tau (q)},$$

where *τ(q)*, *q*th mass exponent, can be defined as^48^,

11 $${\rm{\tau }}(q)=\mathop{\mathrm{lim}}\limits_{\varepsilon \to 0}\frac{log\mu (q,\varepsilon )}{\log (\frac{1}{\varepsilon })}=(1-q){D}_{q},$$

where *D*_*q*_ called the generalized fractal dimensions or Rényi dimensions can be calculated as^48^,

12 $${D}_{q}=\frac{1}{q-1}\mathop{\mathrm{lim}}\limits_{\varepsilon \to 0}\frac{\log \,\mu (q,\varepsilon )}{log\varepsilon }(q\ne 1),$$

13 $${{\rm{D}}}_{1}=\frac{{\sum }_{j=1}^{N(\varepsilon )}{p}_{j}log{p}_{j}}{\log (\varepsilon )}(q=1),$$

## Results and data analyses

### Conventional characteristics

The vitrinite random reflectance (Rr, %) of the coal samples was ranged from 0.58% to 3.43%, corresponding to medium-rank coal (bituminous coals A, B, C, and D) to high-rank coal (anthracite coals B and C) (ISO 11760, 2005)^54^. Proximate analysis showed that the volatility of the coals varied from 5.95% to 43.45%. The moisture content was similar in different coal ranks. The fixed carbon ranged from 27.83% to 80.88%. The total sulfur content changed from 0.42% to 9.36%. The ash yields also had a wide range, varying from 5.47% to 49.09%  (Table 1).

**Table 1:** Properties of the coal samples having different coal ranks.

Samples	Coal coalfield	Rr (%)	Coal ranks	Proximate analysis. %				Ultimate analysis. %				
				M_d_	A_d_	V_d_	FC_d_	S_t.d_	O_daf_	C_daf_	H_daf_	N_daf_
S_1_	Ningwu	0.58	Bituminous D	1.02	49.09	23.07	27.83	9.36	12.36	63.23	4.95	1.08
S_2_		0.63	Bituminous C	1.01	5.47	43.45	51.09	2.51	8.49	81.83	5.66	1.37
S_3_	Pingdingshan	1.31	Bituminous B	1.00	38.81	18.17	43.02	0.42	3.69	80.61	4.43	1.57
S_4_	Dongqu	1.59	Bituminous A	1.01	6.47	14.82	78.79	1.78	4.76	87.88	4.13	1.32
S_5_	Guandi	2.13	Anthracite C	1.01	9.40	9.71	80.88	1.79	3.36	89.73	3.69	1.23
S_6_	Longle	3.08	Anthracite B	1.02	26.61	7.04	66.35	4.94	3.89	86.13	2.52	0.74
S_7_	Fenghuangshan	3.43	Anthracite B	1.01	16.03	5.95	78.02	5.47	0.85	89.28	2.61	0.75

### Macropores structure from HPMI experiments

#### Pore structure distribution

Table 2 lists the pore parameters obtained from the HPMI. In this study, the classification standards defined by Yao *et al*.^28^ and the sharpness of the curve were used. The following three ranges are present: V_1_ < 100 nm, 100 < *V*_*2*_ < 1000 nm, and *V*_3_ > 10000 nm. Samples S_2_ and S_1_ have the largest and smallest cumulative pore volume in *V*_1_, respectively. Cumulative pore volumes *V*_2_ have a relatively lower distribution than *V*_1_ or *V*_3_, and among the samples, sample S_3_ has the highest distribution. The coal rank ranges from bituminous A to anthracite B when the cumulative pore volume, *V*_2_, is the same (0.002 cm^3^/g). Samples S_1_ and S_3_ have the smallest and largest cumulative pore volumes *V*_3_, respectively, which are in response to the total pore volume.

**Table 2:** Results of the HPMI and pore distribution for different coal ranks.

Samples	Cumulative pore volume (cm^3^/g)			Mean volume (cm^3^/g)	Mean surface area (m^2^/g)	Total pore volume (cm^3^/g)
	*V*_1_	*V*_2_	*V*_3_			
S_1_	0.011	0.009	0.006	0.018	0.306	0.026
S_2_	0.039	0.003	0.024	0.030	0.369	0.066
S_3_	0.015	0.014	0.005	*	*	0.033
S_4_	0.024	0.002	0.105	0.115	0.158	0.130
S_5_	0.021	0.002	0.115	0.120	0.062	0.137
S_6_	0.015	0.002	0.075	0.082	0.097	0.092
S_7_	0.019	0.002	0.202	0.020	0.204	0.223

Note: *V*_1_, *V*_2_, and *V*_3_ are the cumulative pore volumes of <100 nm, 100–10000 nm, and >10000 nm respectively, and *V*_*t*_ = *V*_1_ + *V*_2_ + *V*_3_. *no data.

**Figure 2:**
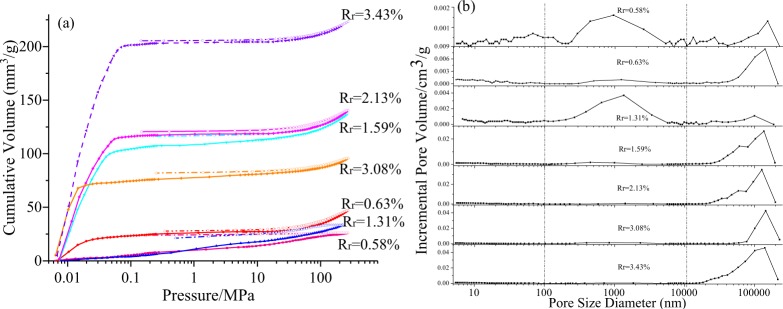
(**a**) Mercury injection and withdraw curves, and (**b**) Pore size distribution.

The mercury injection and withdraw curves as well as the pore size distribution are shown in Fig. 1a,b, respectively. For the high-rank coals, the mercury intrusion and extrusion curves (Fig. 2a) display a similar trend (parallel type), indicating the dominance of the parallel plate pores and a good connectivity for gas diffusion. The shape increases in case of mercury intrusion at low pressures reveal a high proportion of V_3_, and the slightly straight lines reveal a poorly developed V_1_, which are in agreement with pore volume distribution. For the medium-rank coals, there is larger space between the mercury intrusion and extrusion curves (hysteresis loop) (Fig. 2a) than between those of the high-rank coals (tip-edge type), indicating a larger V_1_, small V_2_ and V_3_, and poorer pore connectivity than that in the high-rank coals. The larger hysteresis loop suggests a significant existence of the semi- closed pores. The pore size distribution curves (Fig. 2b) remarkably change with the increase in the coal rank.

#### Fractal dimensions by Sierpinski model

**Figure 3:**
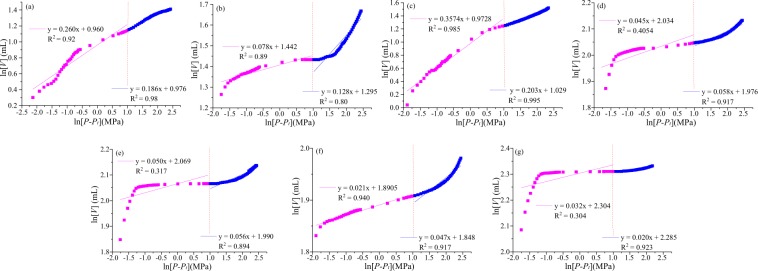
Fractal dimensions D_s2_ and D_s1_ for different coal ranks based on the Sierpinski model.

The fractal curves based on the Sierpinski model are presented in Fig. 3. Fractal dimension *D*_*s1*_ and *D*_*s2*_ are obtained in low-pressure (<20 MPa, responding to the see page pores, >100 nm) and high-pressure (<20 MPa, responding to adsorption pores, <100 nm) stages. The correlation index of *D*_*s1*_ ranges from 0.30 to 0.99. Medium-rank coals S_1_, S_2_ and S_3_ and high-rank coal S_5_ present a better correlation. However, the correlation index of *D*_*s2*_ had a high correlation index (0.80–0.995). The values of *D*_*s1*_ and *D*_*s2*_ ranges from 2 to 3, indicating their power law relationship with the fractal pore surface. The values of *D*_*s1*_ and *D*_*s2*_ are 2.64–2.98 (2.90 in average) and 2.80–2.98 (2.88 in average), respectively.

#### Fractal dimensions by sponge model

**Figure 4:**
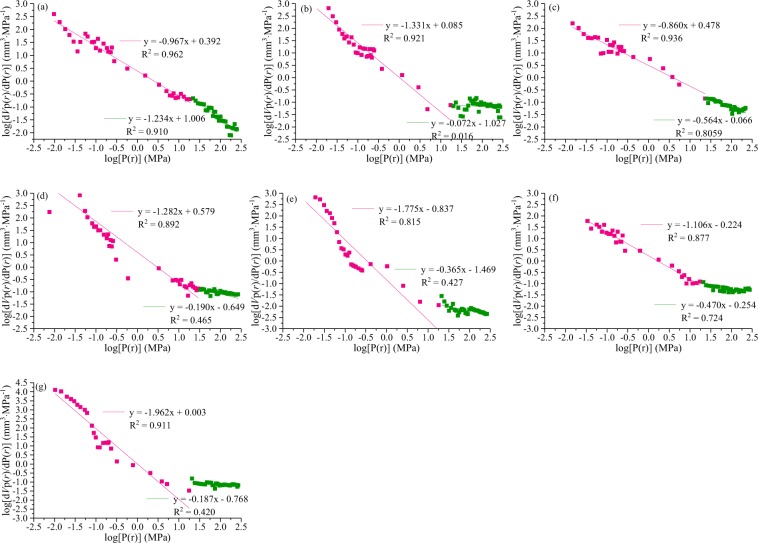
Fractal dimensions *Ds2* and *Ds1* for different coal ranks based on the sponge model.

The fractal curves are divided into two stages based on the classic geometry model (sponge model) (Fig. 4), and thus two fractal dimensions (*D*_*g1*_, low-pressure and *D*_*g2*_, high-pressure) are obtained using the mercury intrusion data. *D*_*g1*_ exhibits a good linear relationship (correlation index *R*^2^, 0.82–0.96), whereas *D*_*g2*_ has a wide range of R^2^ (0.02–0.91). The values of *D*_*g1*_ are widely distributed (2.04–3.14, 2.67 in average), indicating significant differences in the discontinuities and roughness of different coal ranks. However, the value of *D*_*g2*_ are 2.77–3.93 (3.56 in average). Most of the values of *D*_*g2*_ are close to 3, indicating that the surface is extremely rough and the pore structure is irregular^55^. The values of *D*_*g1*_ in S_1_ and S_3_ are >3, and all the values of *D*_*g2*_ are more than 3, except for sample S_1_. For the fractal dimensions of 3, numerous explanations have been provided in previous research^28,44^.

#### Multifractal analysis

**Figure 5:**
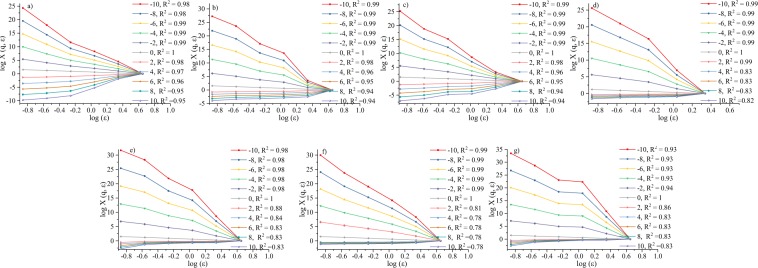
Partition function, logχ(q, ε), box size, and logε for the pore size distributions.

**Figure 6:**
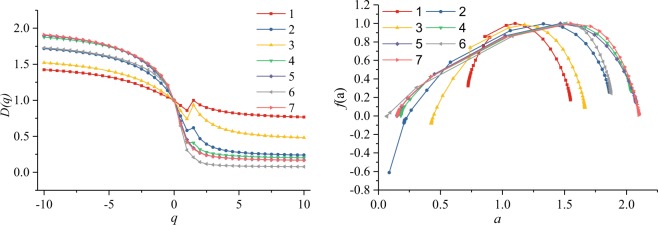
(**a**) Curve of Dq versus q(−10, 10) of the coal samples and (**b**) the multifractal singularity spectra of the coal samples.

The spectrum curves of logχ(q, ε) versus logε show a linear relationship (correlation index *R*^2^, 0.78–1), indicating a multifractal distribution of the pore sizes^56^ (Fig. 5). The spectrum curves of the generalized dimensions, *D(q)*, versus q present a sigma-shaped curve and follow a monotone decreasing function of *q* (Fig. 6a). The characteristic parameters of *D(q)*, dimensions *D*_0_, *D*_1_, and *D*_2_, Hurst exponent *H* (2 *H =D*_*2*_+*1*), width *D*_*−*10_*–D*_*1*0_ of *D(q)* spectrum, right side width *D*_*0*_*–D*_10_, and left side width *D*_*−1*0_*–D*_*0*_ (Table 3), reflect the inner variability and heterogeneity of the porosity and pore size distribution^45,51,57^. Samples S_1_ and S_6_ have the highest and lowest values of *D*_1_, *D*_2_, *H*, and *D*_10_, respectively. The values of *D*_1_ widely range from 0.31 to 0.86.

**Table 3:** Multifractal parameters from the generalized dimension spectrum.

Samples	D_0_	D_1_	D_2_	H	D_10_	D_−10_	D_−10_–D_10_	D_0_–D_10_	D_−10_–D_0_	a_0_
S_1_	1	0.86	0.94	0.97	0.77	1.42	0.66	0.23	0.42	1.10
S_2_	1	0.58	0.47	0.73	0.24	1.72	1.48	0.76	0.72	1.33
S_3_	1	0.74	0.80	0.90	0.48	1.52	1.04	0.52	0.52	1.18
S_4_	1	0.42	0.32	0.66	0.20	1.88	1.67	0.80	0.88	1.51
S_5_	1	0.45	0.27	0.64	0.17	1.90	1.73	0.83	0.90	1.47
S_6_	1	0.31	0.14	0.57	0.08	1.73	1.65	0.92	0.73	1.51
S_7_	1	0.42	0.27	0.63	0.17	1.91	1.74	0.83	0.91	1.55

D_0_, the capacity dimension; D_1_, the entropy dimension; D_2_, the correlation dimension; H, Hurst exponent; D_10_ and D_−10_ are the generalized dimensions responding to q = 10 and q = −10, respectively.

### Propensity to spontaneous combustion

**Figure 7:**
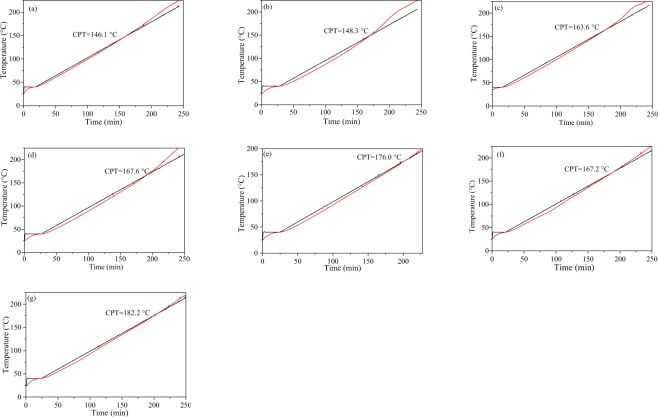
CPT for the different coal ranks.

Fig. 7 displays the results of the CPT measurements performed on the medium-rank and high-rank coals. The CPT ranges from 146.1 to 182.2 °C. Samples S_7_ (anthracite B) and S_1_ (bituminous D) have the highest CPT (182.2 °C) and the lowest CPT (146.1 °C), respectively. The CPT increases with increasing coal rank.

## Discussion

### Effect of evolution of coal petrology on spontaneous combustion

#### Relationship between Rr and CPT and pore structure

**Figure 8:**
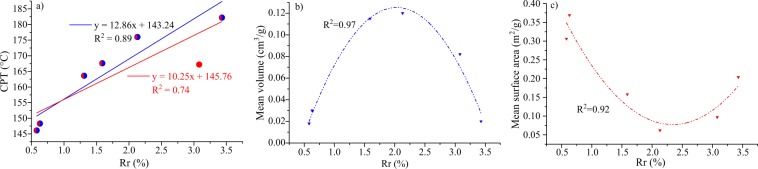
(**a**) Relationship between the vitrinite random reflectance (Rr, %) and (**a**) the CPT, (**b**) mean volume (cm^3^/g), and (**c**) mean surface area (m^2^/g).

Coal rank has a significant influence on the propensity of coal to spontaneous combustion (Fig. 8a). A good linear relationship (R^2^=0.74) is exhibited between the coal rank and the CPT. The CPT increases with increasing coal rank, which is in agreement with previous studies^58–61^. However, sample S_6_ exhibits a deviation compared to the other samples. If this sample data are removed, a better linear (R^2^ = 0.89) is obtained, as shown in Fig. 8a. For the high-rank sample S_6_, the low CPT may be attributed to its different chemical (low fixed carbon) and physical (low total pore volume) structure, which is in agreement with previous studies^11,12^. Coal rank is an important index of coalification, influencing the structures of coal pores and fractures. When the vitrinite random reflectance ranges from 0.58 to 2.13% (Fig. 8b), the mean volume increases with the coal rank, but the mean surface area decreases. When the vitrinite random reflectance ranges from 2.13 to 3.43% (Fig. 8c), the mean volume decreases with coal rank, but the mean surface area increases with coal rank. When the coalification ranges from 0.5 to 2.1%, the aromatic structures including non-protonated aromatic carbons (fa^N^), nuclear magneton resonance (NMR) aromaticity (*f*a’), and aromatic carbon ratio (*f*a) increase linearly, whereas the aliphatic structures decreases linearly^62^. Because the volume is mainly affected by the aliphatic parts of the chemical structure, it may be the cause of the displayed mean volume and surface area trends^63,64^.

#### Relationship between coal composition and CPT

**Figure 9:**
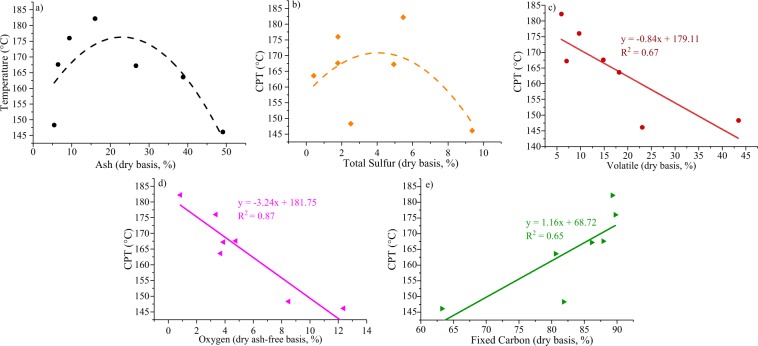
Relationship between the CPT and the (**a**) ash yield, (**b**) total sulfur content, (**c**) volatile matter, (**d**) oxygen content, and (**e**) fixed carbon content.

There exists a non-linear relationship between the ash content and the CPT, which shows an inverted ‘U-shape’ (Fig. 9a). The CPT is a weakly correlated to the total sulfur content (Fig. 9b). The coal components (volatile matter, oxygen contents, and fixed carbon contents) display good relationships with the CPT (Fig. 9c–e), which is in agreement with Zonguldak coals^65^. The CPT decreases with increasing volatile matter, illustrating that low volatile matter is prone to spontaneous combustion. It may be that low volatile matter content can increase the difficulty of ignition and result in an unstable combustion flame^66,67^. The relationship between the oxygen content and the CPT also shows the same trend as the volatile matter, indicating that coals with high oxygen content have a high tendency to chemically bind moisture, thereby rendering the surface, highly susceptible to autogenous heating^68^. The CPT increases linearly with the fixed carbon content, indicating that less fixed carbon content is prone to spontaneous combustion. This is because a small amount of fixed carbon in coal requires a low activation energy to initiate combustion^69^.

### Relationship between pore structure and CPT

#### Distribution of pore structure and CPT

**Figure 10:**
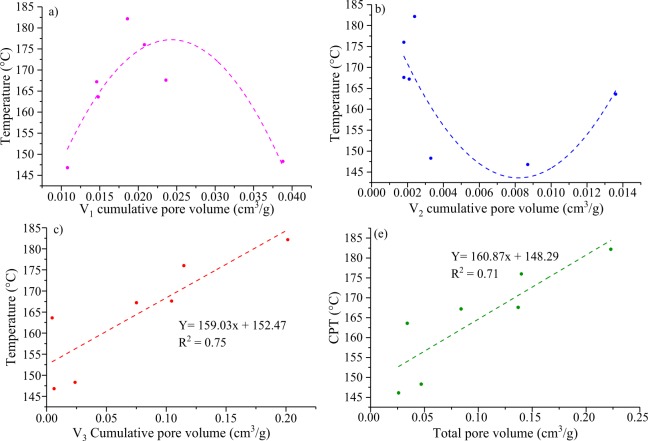
Relationship between CPT and the (**a–d**) pore volume distribution, and (**e**) total pore volume.

It is necessary to discuss the relationship between the pore size and the CPT. Fig. 10a shows a weak negative linear correlation between the cumulative pore volume *V*_1_ and the CPT, which shows an inverted “U-shape”. Cumulative pore volume *V*_2_ shows a “U-shape”(Fig. 10b). The relationship between cumulative pore volume *V*_3_ and the CPT exhibit a linear positive correlation (Fig. 10c). Moreover, the total pore volume also has a good linear positive correlation with the CPT (*R*^2^ up to 0.71), indicating that pore sizes of more than 10000 nm (*V*_3_) play a main role in coal spontaneous combustion (Fig. 10d). Specifically, the pore structure is a dominant factor causing the coal spontaneous combustion, particularly in pore sizes more than 10000 nm.

#### Relationship between coal rank, pore volume, and multifractal parameters

**Figure 11:**
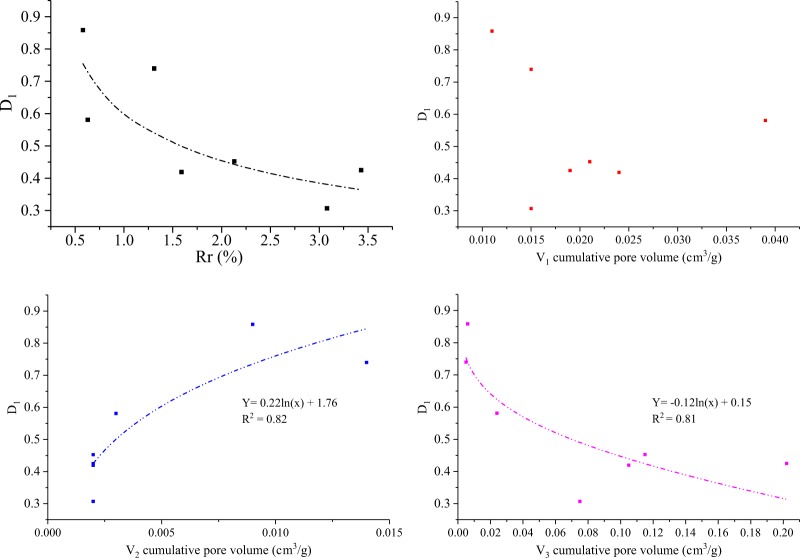
Relationship between *D*_1_ and the (**a**) vitrinite random reflectance (*Rr*, %) and (**b–d**) Cumulative pore volume (*V*_1_, *V*_2_ and *V*_3_).

The entropy dimension (*D*_1_) reveals the concentration degree of the porosity distribution^48^. The values of *D*_1_ are less than or equal to D_0_. When *D*_1_ is close to *D*_0_, the porosity is evenly distributed. Otherwise, most particles are concentrated in a small area and appear as a high peak on the graph^70^. Among of all sample S_1_ has the highest homogeneous pore size distribution. *D*_1_ decreases with increasing coal rank (Fig. 11a), indicating an increase in the heterogeneity. However, the fitting results do not a high correlation.

This may be owing to joint action factors such as maceral content, ash content, volatile matter, and tectonic deformations. The relationship between cumulative pore volume *V*_1_ and *D*_1_ is not clear, indicating that *V*_1_ (adsorption-pores) may have little influence on the entropy dimension (*D*_1_) (Fig. 11b). The *D*_1_ increases with increasing cumulative pore volume *V*_2_ (Fig. 11c) and decreases with increasing cumulative pore volume *V*_3_ (Fig. 11d), indicating that seepage-pores have an important influence on the entropy dimension (*D*_1_).

**Figure 12:**
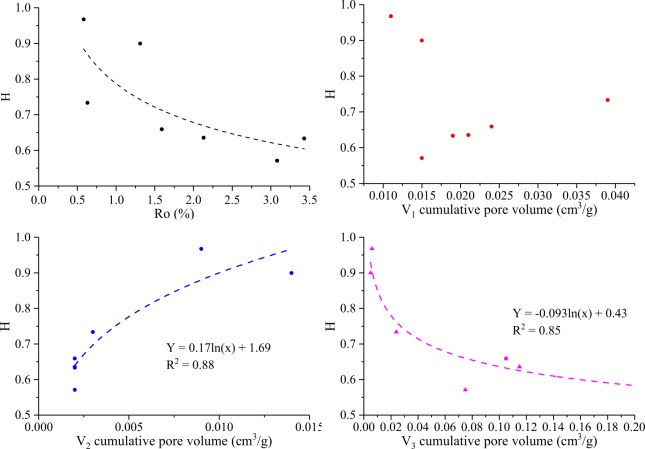
Relationship between the Hurst exponent (*H*) and the (**a**) vitrinite random reflectance (*Rr*, %) and (**b**–**d**) Cumulative pore volumes (*V*_1_, *V*_2_, and *V*_3_).

The Hurst exponent (*H*) is used to quantify the degree of correlation on the logarithmic scale^71^. If *H* > 0.5, the increments are correlated^71^. The Hurst exponent of all the samples exceeds more than 0.5 (H, 0.57–0.97), indicating the increments are correlated (Table 3). The Hurst exponents of samples S_1_ and S_3_ are 0.97 and 0.90, respectively, which two data are close to the 1, reflecting the presence of strong persistence or positive autocorrelations^49,71^. The Hurst exponent has the same characteristics as entropy dimension (Fig. 12).

**Figure 13:**
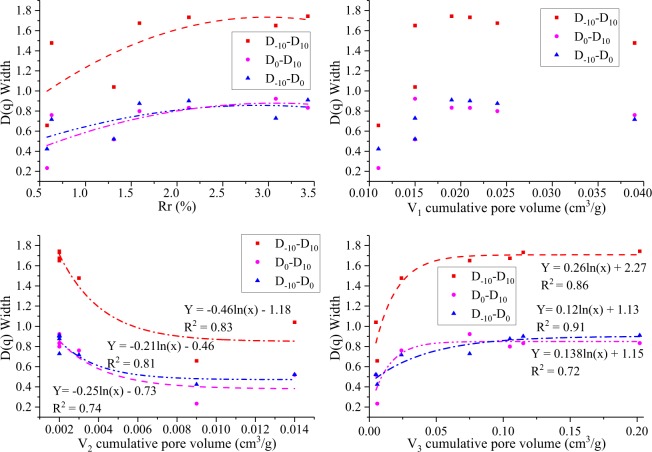
Width of the D (q) spectra (*D*_*−*10_*–D*_*1*0_, *D*_*0*_*–D*_10_, and *D*_*−1*0_*–D*_*0*_) and the (**a**) vitrinite random reflectance (Rr, %) and (**b**–**d**) cumulative pore volumes (V_1_, V_2_, and V_3_).

The width of *D(q)* reflects the heterogeneity in the porosity distribution^45,51^. Sample S_7_ has the highest *D*_*−*10_ − *D*_10_ (the widest spectrum), indicating the highest heterogeneity over the entire pore size range among all the coal samples^45,51^. In contrast, sample S_1_ has the lowest *D*_*−10*_*-D*_*10*_ (the narrowest spectrum), reflecting the lowest heterogeneity in the porosity distribution over the entire pore size range^45,51^. Samples S_2_ and S_6_ have wider right side *D(q)* spectra than the left side *D(q)* spectra, indicating high dominance of the high porosity concentrations^45^. However, samples S_1_, S_4_, S_5_, and *S*_7_ have wider left side *D(q)* spectra than right side *D(q)* spectra, indicating a small porosity concentration^45^. The widths of the *D(q)* (*D*_*−*10_*–D*_*1*0_, *D*_*0*_*–D*_10_, and *D*_*−1*0_*–D*_*0*_) spectra increase with increasing vitrinite random reflectance (*Rr*, %) (Fig. 13a). The relationship between cumulative pore volume *V*_1_ and width of the *D(q)* spectra is not clear, indicating that *V*_1_ (adsorption-pores) may have little influence on the width of the *D(q)* spectra (Fig. 13b). The width of the *D (q)* spectra logarithmically decreases with increasing cumulative pore volume *V*_2_ (Fig. 13c) and logarithmically increases with increasing cumulative pore volume *V*_3_ (Fig. 13d). The above indicates that seepage-pores have an important influence on the heterogeneity in the porosity distribution.

#### Fractal dimensions and CPT

##### 1) Multiscale fractal dimensions and CPT

**Figure 14:**
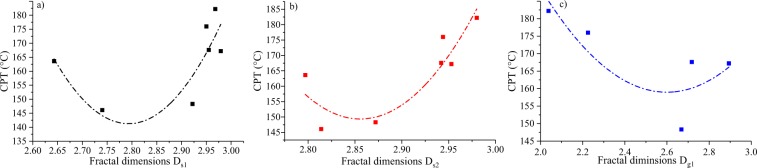
Relationship between the CPT and (**a**) fractal dimensions *D*_*s1*_, (**b**) fractal dimensions *D*_*s2*_, and (**e**) fractal dimensions *D*_*g1*_.

The relationships between the fractal dimensions of the Sierpinski model (*D*_*s*1_ and *D*_*s*2_) and the CPT are shown in Fig. 11. The relationship between *D*_*s1*_ and the CPT can be divided into two distinct stages (Fig. 14a). In the first stage, the CPT first decreases, and then increases with increasing *D*_*s1*_ (when *D*_*s1*_ > 2.8). With increasing *D*_*s2*_, the CPT decreases first (from 2.75 to 2.85), and then increases (Fig. 14b). Fractal dimensions *D*_*g1*_ of samples S_1_ and S_3_ are >3; however, most of the fractal dimensions, *D*_*g2*_, are >3, except of sample S_1_. Fractal dimensions *D*_*g1*_ and *D*_*g2*_ are >3, which are not suitable to characterize the pore heterogeneity^72,73^. To prevent the interference of abnormal points, the data for fractal dimensions >3 were removed^37^. The relationship between *D*_*g1*_ and the CPT (without samples S_1_ and S_2_) displays a similar trend as that between *Ds* and the CPT obtained from the Sierpinski model (Fig. 14c). When *D*_*g1*_ ranges from 2 to 2.6, the CPT decreased significantly. When *D*_*g1*_ becomes larger than 2.6, the CPT increases with increasing *D*_*g1*_. The above results demonstrate that the heterogeneities obtained from the Sierpinski and Sponge models do not present linear relationship with the tendency of coal to spontaneous combustion. However, a high heterogeneity (fractal dimensions >2.8) is associated with a low tendency of spontaneous combustion.

##### 2) Multifractal fractal dimensions and CPT.

**Figure 15:**
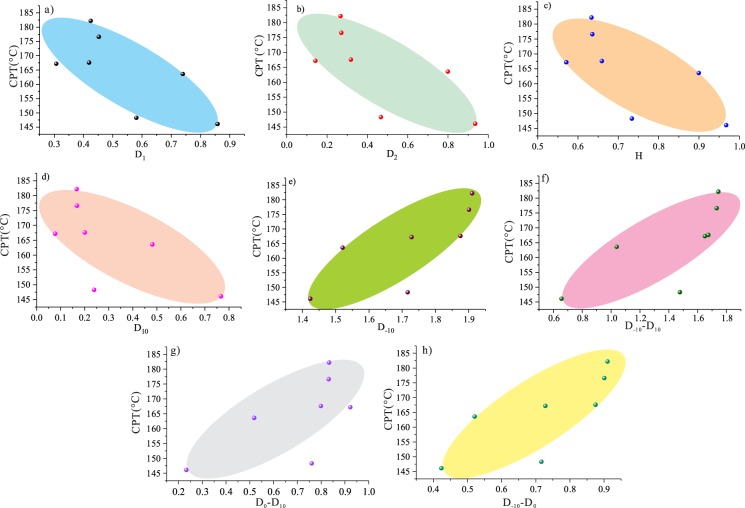
Relationship between the CPT and the multifractal parameters.

Fig. 15 displays the correlation between the multifractal fractal dimensions (*D*_*1*_, *D*_*2*_, *H*, *D*_10_, *D−*_10_, *D*_*−*10_*-D*_*1*0_, *D*_*−1*0_*-D*_*0*_, *D*_*0*_*-D*_10_) and the CPT. It can be found that the CPT decreases with increasing parameters *D*_1_, *D*_2_, *H*, and *D*_10_, suggesting that a high degree of the distribution of the porosity quantifies the degree of correlation on the logarithmic scale. Further, the heterogeneity in the porosity distribution decreases the tendency of coal spontaneous combustion. The correlation between *D*_*−*10_, *D*_*−*10_*-D*_*1*0_, *D*_*−1*0_*-D*_*0*_, and *D*_*0*_*-D*_10_ and the CPT is clear in that the CPT gradually increases with increasing *D*_*−*10_*-D*_*1*0_, *D*_*−1*0_*-D*_*0*_, and *D*_*0*_*-D*_10_. This indicates that the complexity of the local characterization pore structure decrease the spontaneous combustion propensity.

## Conclusions

The conventional analysis and CPT measurements were conducted to obtain the properties of coal petrology and spontaneous combustion. Coal rank, volatile matter, oxygen content, and fixed carbon content were found to play important roles in spontaneous combustion.

The pore structure properties obtained from HPMI provided a direct measurement of coal physical properties. The cumulative pore volume of *V*_3_ (>10000 nm) and total pore volume have positive correlation with CPT.

Multiscale and multifractal analyses were conducted to evaluate the pore size distribution. From the multiscale analysis, the relationship between the fractal dimensions (D_s1_, D_s2_, and D_g1_) and the CPT basically displayed a “U-shaped” tendency, with the minimum occurring at 2.6–2.85. Based on the multifractal analysis, a high degree of porosity distribution, quantified the degree of correlation on the logarithmic scale. Furthermore, the heterogeneity in the porosity distribution decreased the tendency of coal spontaneous combustion; therefore, a more complex local characterization pore structure lowered the spontaneous combustion propensity.
